# Genome-Wide Identification of Genes Related to Biosynthesis of Phenolic Acid Derivatives in *Bletilla striata* at Different Suspension Culture Stages

**DOI:** 10.3389/fpls.2022.875404

**Published:** 2022-06-17

**Authors:** Houbo Liu, Ceyin Huang, Qingqing Li, Mufei Wang, Shiji Xiao, Junhua Shi, Yihuai He, Weie Wen, Lin Li, Delin Xu

**Affiliations:** ^1^Department of Cell Biology, Zunyi Medical University, Zunyi, China; ^2^Department of Dermatology, Chengdu Second People's Hospital, Chengdu, China; ^3^School of Pharmacy Chemistry, Zunyi Medical University, Zunyi, China; ^4^Affiliated Hospital of Zunyi Medical University, Zunyi, China

**Keywords:** *Bletilla striata*, phenolic acid derivatives, suspension culture system, biosynthesis, regulate genes

## Abstract

To screen the genes regulating the biosynthesis of phenolic acid derivatives from the genome of *Bletilla striata*, we designed a suspension culture system to sample the cells for the following experiments. The contents of four phenolic acid derivatives were determined by high-performance liquid chromatography, and several full-length transcriptome sequencings of RNA samples at 10 time points were performed for bioinformatics analysis. The correlation analysis was used to identify and verify the key DEGs involved in the biosynthesis of the four phenolic acid derivatives. The results showed that the contents of p-hydroxybenzylalcohol (HBA), Dactylorhin A, Militarine, and Coelonin peaked at 33 days postinoculation (Dpi), 18 Dpi, 39 Dpi, and 39 Dpi of the culture system, respectively. Based on transcriptome data, 80 DEGs involved in the biosynthesis of phenolic acid derivatives were obtained. The KEGG pathway enrichment analysis classified them mostly into five metabolic pathways: phenylpropane biosynthesis, starch and sucrose metabolic, cyanoamino acid metabolism, gluconeogenesis and glycolysis, and phenylalanine metabolism. qPCR analysis revealed that the relative gene expression levels were consistent with the overall trend of transcriptome sequencing results. Among them, 14, 18, 23, and 41 unigenes were found to be involved in the synthesis of HBA, Dactylorhin A, Coelonin, and Militarine, respectively. These unigenes laid a solid foundation for elucidating the biosynthesis mechanism of phenolic acid derivatives in suspension cells of *B. striata*.

## Introduction

*Bletilla striata* is a perennial herb of *Orchidaceae* andalso a traditional and precious Chinese herbal medicine with a high concentration of medicinal metabolites. For thousands of years, it has been prescribed in traditional Chinese medicine to treat hematemesis and promote wound healing (Zhao et al., 2021). A recent report detailed that *B. striata* has therapeutic effects on gastrointestinal bleeding, hemoptysis, traumatic bleeding, and postpartum hemorrhage (Zhang et al., 2022). Phenolic acid derivatives are a class of compounds with a polyhydroxyphenol structure, which are important secondary metabolites for supporting most pharmacological activities, such as antioxidant, anti-tumor, and anti-inflammatory, of *B. striata* (Kassim et al., 2010; Anantharaju et al., 2016; Nigro et al., 2017; Milovanović et al., 2021; Yuan et al., 2021). Among the metabolites, p-hydroxybenzylalcohol (HBA), Dactylorhin A, Millitarine, and Coelonin were the most abundant active components in *B. striata*. In our previous study, we found that the content of these secondary metabolites could reach 0.793 mg/g, 7.792 mg/g, 9.447 mg/g, and 0.345 mg/g of dried suspension cells, respectively (Pan, 2019). A number of studies further demonstrated that HBA has anti-inflammatory, anti-oxidation, anti-convulsion, anti-depression, and ameliorating effects on memory impairment, as well as protective benefits against brain damage caused by cerebral ischemia and sedative and hypnotic effects (Luo et al., 2017; Ding et al., 2019; Zhang D. et al., 2020). In recent years, many kinds of research have further confirmed the anti-cancer and antioxidant properties of dihydrophenanthrene compounds (Boudjada et al., 2019; Jiang et al., 2019). In conclusion, HBA, Dactylorhin A, Millitarine, and Coelonin are natural chemicals with the potential for drug development.

However, a variety of phenolic acid derivatives have been identified in species such as *B. striata, Flickingeria fimbriata*, (Wu et al., 2017) and *Dendrobium scabrilingue* (Sarakulwattana et al., 2020), but their biosynthetic mechanisms and the synergistic regulation mechanisms of multiple genes are still unclear. Therefore, it is important to find the genes that regulate the biosynthesis of secondary metabolites through genetic improvement way or engineering bacteria.

Former studies found that the biosynthesis pathways of phenolic acids mainly include the shikimic acid metabolic pathway, the phenylpropanoid metabolic pathway, and the flavonoid metabolic pathway (Marchiosi et al., 2020). However, the biosynthetic pathways of phenolic acid derivatives have not been clearly explained. By analyzing the metabolic pathways of key time nodes related to HBA, Dactylorhin A, Millitarine, and Coelonin, we see that they may provide a theoretical basis for exploring the synthetic methods for phenolic acid derivatives.

In this study, aided by the full-length transcriptome sequencing of PacBio Sequel and Illumina short-read sequencing technology (Zhou et al., 2021), we first profiled gene expression in different growth stages of the lag phase, exponential phase, deceleration phase, and decline phase of the suspension culture system of *B. striata*. Then, we screened out the genes involved in the biosynthesis of phenolic acid derivatives. Finally, we verified the expression patterns of selected genes in the suspension system. The obtained conclusions may provide a theoretical foundation for the genetic breeding of *B. striata* as well as an experimental basis for exploring the molecular biosynthesis mechanism of phenolic acid derivatives.

## Materials and Methods

### Material

The capsules were harvested from the *B. striata* Germplasm Garden of Zunyi Medical University, Xinpu District, Zunyi City, Guizhou Province, China (27°42' N, 107°01' E). After disinfection (Li et al., 2020), matured seeds obtained from capsules were induced for the following suspension culture. The information on the compound standards used for measuring the contents of phenolic acid derivatives is shown in Supplementary Table S3. In addition, the compound structures are shown in Supplementary Figure S1.

### Methods

#### Culture of Suspension Cells

Suspension culture was carried out for the efficient biosynthesis of phenolic acid by selecting *B. striata* with relatively uniform growth and good induction. Based on the contents of various compounds, we used MS (Murashige & Skoog Medium) as the base medium, with the addition of 1.0 mg/L of 6-BA, 3.0 mg/L of 2,4-D, and 30.0 g/L of sucrose. The pH was adjusted to 5.8–6.0. After 45 days of induction and 30 days of subculture, the content of secondary metabolites of suspension-cultured cells was measured every 3 days for 45 days postinoculation (Dpi). In addition, the content of phenolic acid derivatives was calculated according to the standard curve (Pan, 2019). The suspension-cultured cells were stored at 3 Dpi, 9 Dpi, 18 Dpi, 27 Dpi, 30 Dpi, 33 Dpi, 36 Dpi, 39 Dpi, and 42 Dpi for measuring the chemical content and RNA-Seq sequencing as described below.

#### Content Determination of Phenolic Acid Derivatives

Starting from the day of the directional suspension culture, the contents of phenolic acid derivatives were measured every 3 days, and the sampling was repeated three times. After being drained and dried, the cell clusters were crushed with a mortar, and 0.2 g was accurately weighed and placed in a round-bottom flask. Then, the cells were heated and refilled with 70% methanol-water for 2 h, transferred to a 5-ml volumetric flask filled with 70% methanol-water to maintain a constant volume, shaken well, and filtered using a 0.45 μm filter membrane for subsequent experiments. The content of phenolic acid derivatives in cells was determined by HPLC (Waters e2695).

The chromatographic conditions and gradient elution conditions were as follows: Chromatographic conditions: column: Universil C18 column (250 mm×4.6 mm, 5 μm, Kromat Corporation); other conditions were listed in Supplementary Table S4.

#### Transcriptome Data Assembly and Sequencing

After grinding the selected callus materials of 10 time points with liquid nitrogen, total RNA was extracted by Trizol and stored at −80 °C for subsequent sequencing on PacBio Sequel and Illumina Hiseq 2500 platforms, which were entrusted to us by Novogene Co., Ltd. To obtain clean reads, high-throughput sequencing from a large amount of raw data was obtained, from which the adaptor sequence, primer sequence, and low-quality reads were removed. The LoRDEC (Salmela and Rivals, 2014) software was used to correct the PacBio Sequel data with the Illumina Hiseq 2500 data. The CD-HIT (Fu et al., 2012) software was used to de-redundant the corrected consensus sequence, and the obtained transcript set was applied as the reference sequence (ref) for subsequent analysis. Then, the clean reads of each sample obtained by Illumina sequencing were compared to the reference to determine the transcripts detected in each sample.

#### Screening of DEGs

Using the RSEM (Li and Dewey, 2011) software to carry out the statistical analysis on the comparison results of Bowtie2 and perform FPKM conversion to determine the expression levels of the genes detected in the samples. The resulting q-values were adjusted by Benjamini and Hochberg's method. DEGs among different samples were screened based on |log_2_(FoldChange)|≥ 2 and a *q-*value < 0.05.

#### GO Enrichment and KEGG Pathway Analysis of DEGs

We used GO (http://www.geneontology.org/) and KEGG databases (http://www.genome.jp/kegg/) to perform functional enrichment analysis on the selected DEGs. It was considered that the GO function or KEGG pathway was significantly enriched (*q*-value < 0.05), and the detection was annotated to DEGs in different GO functional groups or metabolic pathways.

#### Screen the key Genes Involved in Regulating the Synthesis of Phenolic Acid Derivatives

Using the K-means cluster analysis method, the DEGs with similar expressions were grouped into one category, and the differential gene expression trend was analyzed. Using the R package to analyze the correlation between DEGs and phenolic acid derivatives of HBA, Dactylorhin A, Militarine, and Coelonin at 10 time points (Spearman correlation coefficient), the screening criteria were *q-*value < 0.05 and R > 0.05. Subsequently, according to the accumulation and change curves of the four compounds on different days, DEGs at different time points were analyzed by the KEGG pathway and GO function analysis. The DEGs with significant upregulation or downregulation were screened. The main metabolic pathways involved in DEGs were determined. Finally, combined with correlation analysis, the key genes involved in the synthesis of phenolic acid derivatives were screened.

#### QPCR Verification of DEGs

The detected DEGs closely related to each derivative were randomly selected for verification via qPCR. Approximately 1 μg total RNA was used as the template for synthesizing the first cDNA strand by reverse transcription. The SYBR PrimeScriptTM RT-PCR Kit (TaKaRa) fluorescence quantitative kit was used to carry out qPCR reactions on an AGS 4800 real-time qPCR instrument (Hangzhou Anyang Technology Co., Ltd., Hangzhou, China). Primer Premier software was used to design specific primers for qPCR reactions of different randomly selected genes (Table 1). The approaches for cDNA reverse transcription and the qPCR reaction system are detailed in Supplementary Tables S5, S6. Besides, the cDNA reverse transcription reaction conditions were 42 °C for 60 min and 95 °C for 3 min. In addition, qPCR reaction conditions were enzyme activation at 95°C for 5 min; denaturation at 95°C for 5 s, and extension at 60°C for 30 s, 45 cycles.

**Table 1:** The information of qPCR primers.

**Gene**	**Primer name**	**Primer**	**Primer length (nt)**	**Product length (bp)**	**Tm (**°**C)**
*NA1*	NA1-F	ACGGAGGCGGAGGCAACTAC	20	119	63.12
	NA1-R	GCAGCAGCCGTGGTAGCAAC	20		63.04
*NA2*	NA2-F	GCAGCGGCGGAATCTCTTCG	20	82	62.49
	NA2-R	TGCTGTGGAGCGGGATGACC	20		63.51
*NA3*	NA3-F	CTGGTGTGCGAATGTTGCTTCATC	24	148	59.24
	NA3-R	GCTTTCACGTATCAGGCGTTTGC	23		59.61
*NA4*	NA4-F	GCAATGGGCTACAGGACCAGAAG	23	80	60.74
	NA4-R	TCCGCCGCCTCCGTGATAAC	20		63.34
*C4H1*	C4H1-F	GCGGCGGTGACTCTTGCTATC	21	138	61.64
	C4H1-R	GGTGGCTAGATTGCGGTGATTGAG	24		60.6
*PAL1*	PAL1-F	CCAGACTCGCCATTGCTGCTATC	23	149	60.88
	PAL1-R	GGCTATCTCTGCTCCCTTGAAACC	24		60.05
*PAL2*	PAL2-F	GCTCCTTGCAAGAGTCGATTT	21	117	55.40
	PAL2-R	CAGGCAGAGTCCCTCCATTA	20		56.50
*BGLU1*	BGLU1-F	CACCATCCCATAGCCCGTTTCTAC	24	139	60.04
	BGLU1-R	TCAACTCCCGTAGCAAAGCCATTC	24		59.76
*BGLU2*	BGLU2-F	GAAGGACGGTAGCACAGCAGATG	23	145	60.53
	BGLU2-R	CACCGCTCCTCTCCCTCTTGG	21		63.22
*BGLU3*	BGLU3-F	CGGCTCTCAGGGATGGCTCAG	21	140	63.51
	BGLU3-R	CTTTGGCACTCTCTCACAGGCTTC	24		60.48
*actin*	actin-F	AATCCCAAGGCAAACAGA	18	-	51.00
	actin-R	CACCATCACCAGAATCCAG	19		53.00

#### Data Statistical Analysis

Each experiment was conducted with three biological replicates and three technical replicates. The 2^−Δ*ΔCt*^ method was used to analyze gene expression levels in qPCR arrays. The statistical analysis of the data was run in SPSS, and the statistical methods of the one-way ANOVA and multiple comparisons were applied to the harvested data.

## Results

### Determination of the Content of Phenolic Acid Derivatives in Suspension Cells of *B. striata*

**Figure 1:**
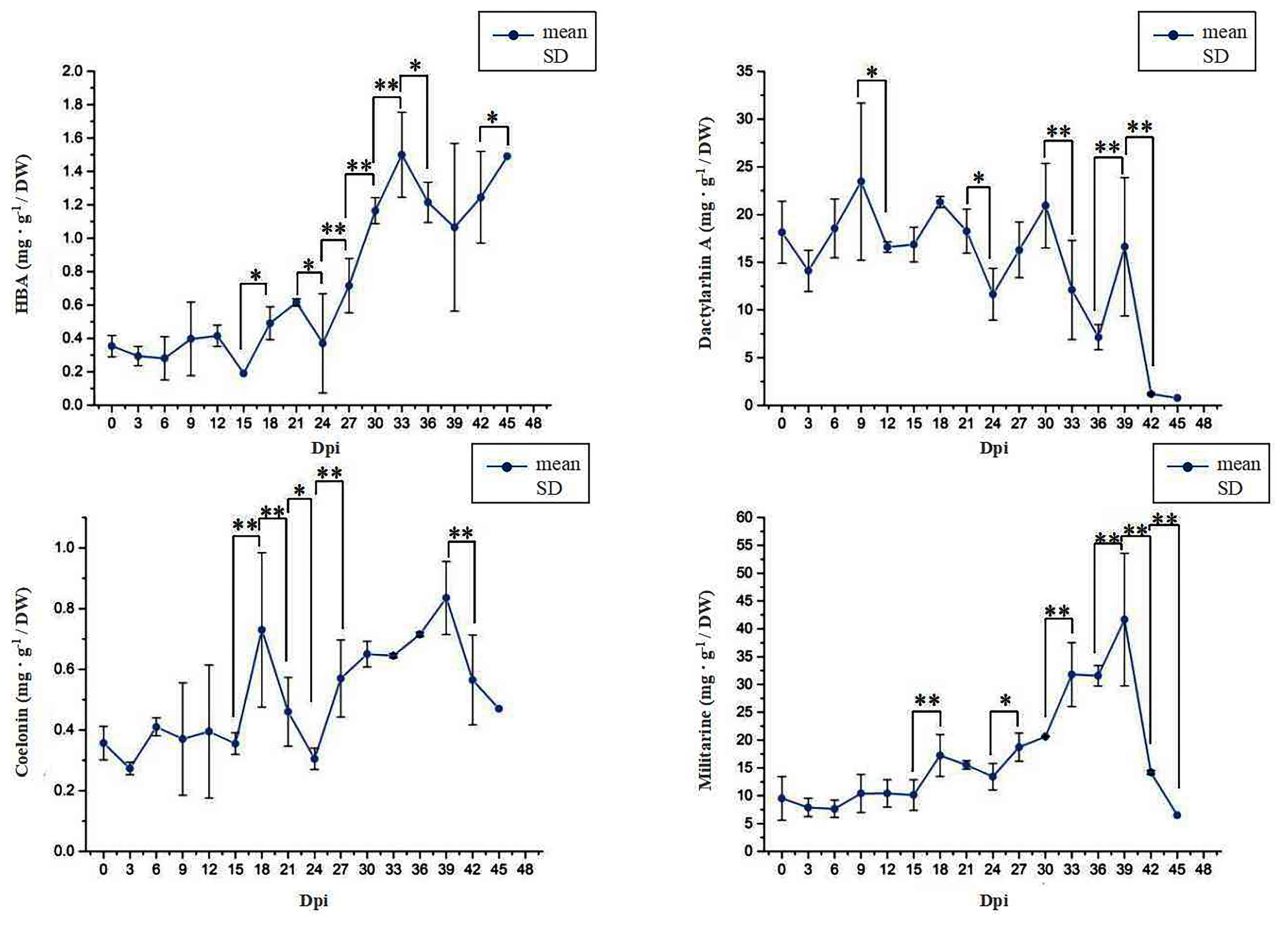
The content changes of four phenolic acid derivatives. * Represents the significant difference between the two groups compared with the previous group at a level of *P* < 0.05. ** represents the significant difference at a level of *P* < 0.01.

The content of the four metabolites varied significantly in different growth stages of suspension culture cells (Figure 1). With the growth of culture, the accumulation of HBA in the suspension culture cells increased first and then decreased before finally reaching the maximum peak at 33 Dpi. The accumulation of Dactylorhin A gradually reduced as the number of days increased and then decreased sharply after 30 Dpi. The accumulation of Militarine peaked at 39 Dpi and then reduced. The accumulation of Coelonin first increased and then decreased with the increase of time, reaching its peak at 39 Dpi. According to the changing trend of the content of different compounds, we selected 18 Dpi, 27 Dpi, and 33 Dpi for HBA; 18 Dpi, 36 Dpi, and 42 Dpi for Dactylorhin A; 3 Dpi, 30 Dpi, 33 Dpi, and 39 Dpi for Militarine; and 3 Dpi, 30 Dpi, 33 Dpi, and 39 Dpi for Coelonin for subsequent experimental analysis.

### Transcriptome Sequencing Analysis of Suspension Cells of *B. striata*

By performing full-length transcriptome sequencing on suspension cells of *B. striata* at different time points, a total of 50.02 Gb of raw data were obtained, which were deposited in the SRA database under the accession number SRR18045794. A total of 902,688 CCS (Circular Consensus Sequence) readings were obtained, including 719,981 reads with 5'- primers, 785,110 reads with 3'- primers, and 781,562 reads with Poly A tail. There were 620,018 full-length (FL) reads. The number of full-length non-chimeric reads (FLNC) was 472,211, and the average length of FLNC was 2,423 bp. The FLNC sequence of the same transcript was converted using the ICE algorithm Clustering, which was performed to eliminate redundancy, and a total of 246,933 identical transcripts were obtained. Using CD-HIT to perform de-redundancy analysis on high-quality transcripts, a total of 100,276 transcripts were obtained, which were used as the final transcript sequence for further analysis (Li et al., 2020).

### Analysis of DEGs at Various Time Points

#### Distribution of DEGs

**Figure 2:**
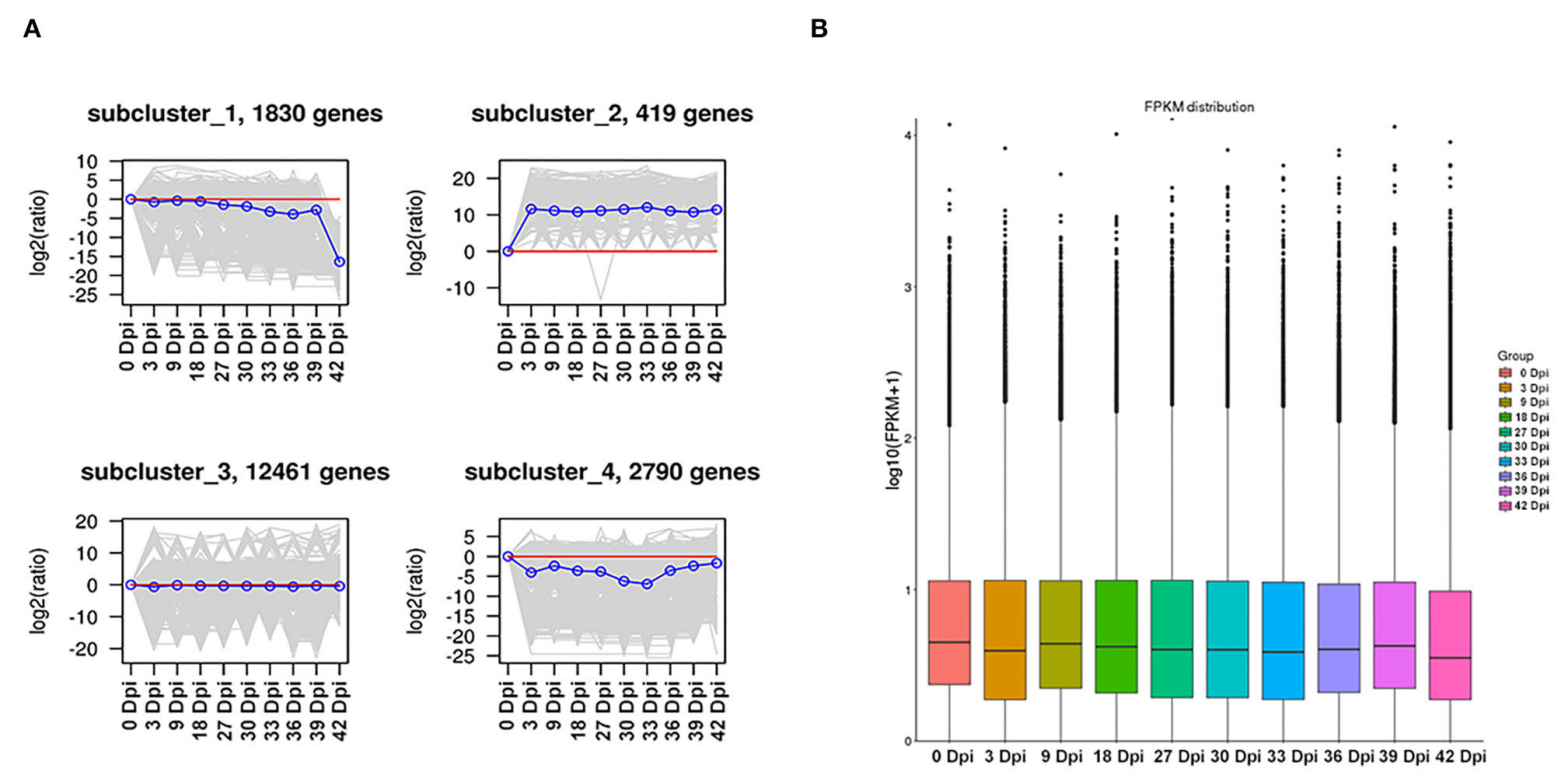
Distribution of DEGs at different time points. **(A)** Expression trend of DEGs by K-means. **(B)** FPKM box plots of different samples.

In order to explore the expression trend of DEGs during the development of suspension cells of *B. striata*, we conducted a K-means analysis on DEGs, which resulted in four gene expression trend graphs (Figure 2A). Although gene cluster 3 contained the largest number of genes, the changes in expression levels in each period were not obvious. The gene expression levels of gene cluster 2 were upregulated throughout the development of suspension cells of *B. striata*, whereas gene clusters 1 and 4 were reduced.

The FPKM value was used to quantify gene expression, and it can be seen from the results that there is little difference in gene expression levels among different samples (Figure 2B). This showed that, after FPKM quantification, the analysis results of DEGs between each sample are accurate and reliable. Subsequently, DESeq2 software was used to analyze the significant difference in the DEGs between different samples, and pairwise comparisons of suspension cells at eight time points were made. It was found that the number of DEGs gradually increased during the growth and breeding of suspension cells. A total of 2,052 DEGs were selected from the 3 Dpi vs. 30 Dpi comparison, which was significantly higher than the other comparisons. In the following comparison groups (3 Dpi vs. 30 Dpi, 18 Dpi vs. 36 Dpi, 27 Dpi vs. 33 Dpi, 36 Dpi vs. 42 Dpi, 30 Dpi vs. 33 Dpi, 39 Dpi vs. 42 Dpi), the number of upregulated genes was significantly higher than that of downregulated genes. In the other three comparison groups (18 Dpi vs. 27 Dpi, 30 Dpi vs. 39 Dpi, 33 Dpi vs. 39 Dpi), the number of upregulated genes was significantly lower than that of downregulated genes. These results indicated that DEGs were closely involved in the synthesis of phenolic acid derivatives in the suspension culture cells of *B. striata*, reflecting the obvious spatio-temporal differences in the synthesis of secondary metabolites.

**Figure 3:**
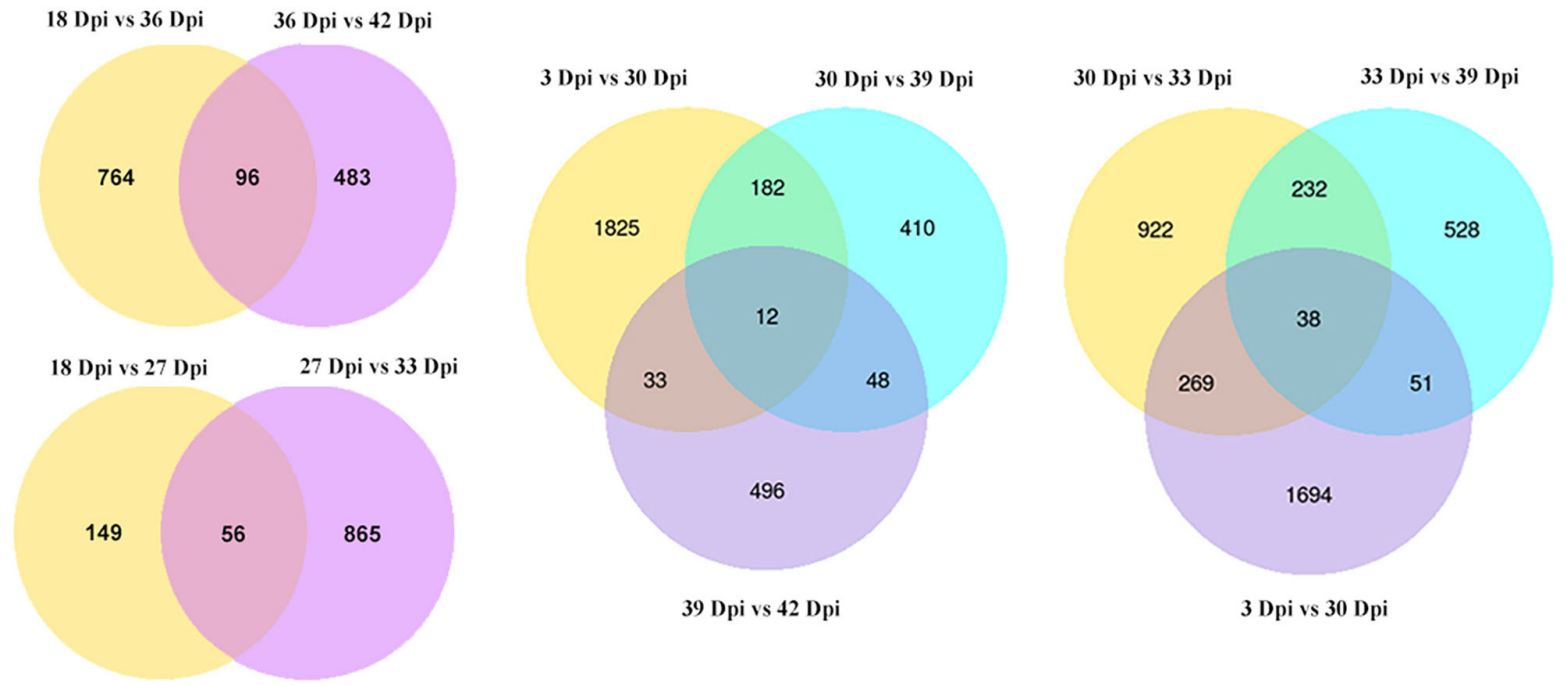
Number of DEGs between different combinations.

We analyzed some comparisons of different time points. We found that there were 56 (18 Dpi vs. 27 Dpi vs. 33 Dpi), 96 (18 Dpi vs. 36 Dpi vs. 42 Dpi), 12 (3 Dpi vs. 30 Dpi vs. 39 Dpi vs. 42 Dpi), and 38 (3 Dpi vs. 30 Dpi vs. 33 Dpi vs. 39 Dpi) DEGs in different time groups, respectively (Figure 3), indicating that these DEGs may play an important role in the growth and development of *B. striata*.

**Figure 4:**
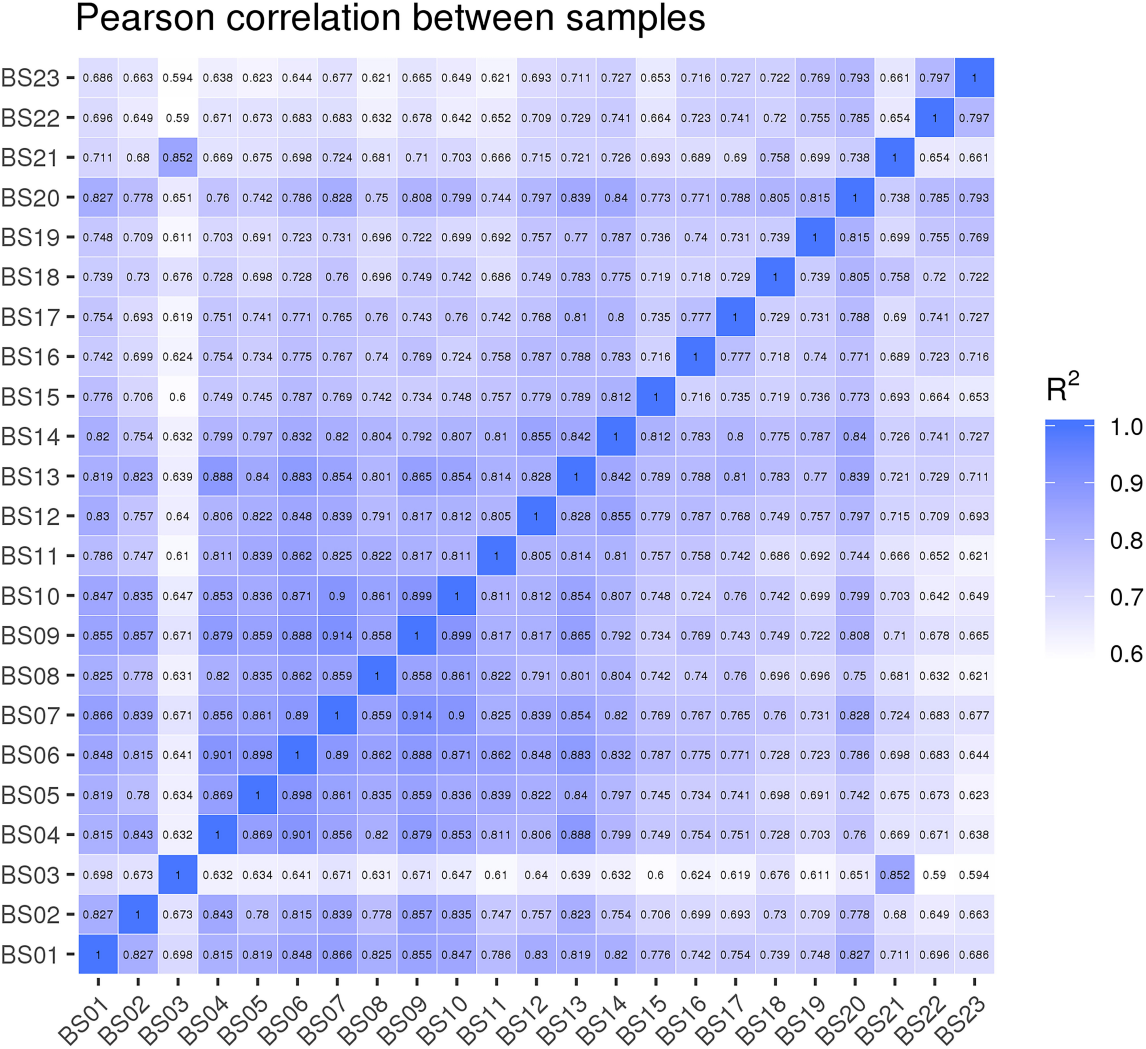
Correlation analysis among samples.

The results of the Pearson correlation analysis showed that the biological duplication between each group was highly correlated. During the development of suspension cells, the correlation values (R^2^) of the following groups were higher than the other comparison groups: 3 Dpi vs. 9 Dpi (R^2^ = 0.861), 3 Dpi vs. 18 Dpi (R^2^ = 0.845), 3 Dpi vs. 27 Dpi (R^2^ = 0. 0.848), 9 Dpi vs. 18 Dpi (R^2^ = 0. 854), 9 Dpi vs. 27 Dpi (R^2^ = 0. 0.828), 18 Dpi vs. 27 Dpi (R^2^ = 0. 822), 18 Dpi vs. 30 Dpi (R^2^ = 0. 816). In the process of growth and development, R^2^ > 0.8 indicated that the differences in the expression of related genes between the different periods were small (Figure 4).

#### DEGs Screening Involved Biosynthesis of Phenolic Acid Derivatives in Suspension Cells

##### Correlation Analysis Between DEGs and Four Main Secondary Metabolites at Each Time Point

We detected correlations between the four phenolic acid derivatives and 17,500 DEGs. Among them, HBA, Dactylorhin A, Militarine, and Coelonin were significantly correlated with 5,134, 3,567, 1,687, and 599 DEGs, respectively (*P* < 0.05) (Supplementary Table S1).

##### DEGs Analysis of HBA

Based on the change in HBA content, we found that it increased gradually from 0 Dpi to 27 Dpi, and then the HBA content began to increase exponentially, where it stabilized at 33 Dpi. Three time points (18 Dpi, 27 Dpi, and 33 Dpi) with significant differences in HBA content were selected for differential expression analysis. At the 18 Dpi vs. 27 Dpi time point, the number of upregulated genes was 78, and the number of downregulated genes was 127. At the 27 Dpi vs. 33 Dpi time point, the number of upregulated genes was 712, while the number of downregulated genes was 209.

The functional clustering arrays of GO and KEGG on the DEGs imply a mechanism for explaining how HBA was biosynthesized. GO function analysis found that, at the 18 Dpi vs. 27 Dpi time point, DEGs were mainly enriched in the binding process, cellular process, metabolic process, and catalytic activity. At the 27 Dpi vs. 33 Dpi time point, DEGs were mainly enriched in the hydrolase activity and nucleoside phosphatase activity. A KEGG pathway analysis of DEGs at three time points found that a total of 39 DEGs were significantly enriched in four pathways (*q-*value < 0.05), namely Circadian rhythm-plant, Cyanoamino acid metabolism, starch and sucrose metabolism, and Phenylpropanoid biosynthesis pathways. At the 18 Dpi vs. 27 Dpi time point, there were 6 DEGs involved in the circadian rhythm of plants (ko04712). At the 27 Dpi vs. 33 Dpi time point, 30 DEGs were significantly enriched in the metabolic pathways of starch and sucrose (ko00500); 18 DEGs were significantly enriched in the cyanoamino acid metabolism pathway (ko00940); and 16 DEGs were enriched in the cyanogen amino acid metabolism pathway (ko00460) (Supplementary Table S2). We selected the pathways of phenylpropanoid biosynthesis and cyanoamino acid metabolism, which are closely involved in the synthesis of HBA. There were 11 upregulated genes with significant differences in the phenylpropane metabolic pathways, encoding β-glucosidase (BGLU) and peroxidase (PRX); there were seven downregulated genes encoding phenylalanine ammonia-lyase (PAL) and BGLU,. In the cyanoamino acid metabolism pathway, the up and downregulated genes with bigger differences were involved in encoding BGLU. Compared with the results of correlation analysis, 18 unigenes (8 enzymes) were finally screened out as being potentially involved in HBA synthesis (Table 2).

**Table 2:** Genes involved in the biosynthesis of secondary metabolites in suspension cells of *Bletilla striata*.

**Secondary metabolites**	**Metabolic pathway**	**Entry ID**	**Key enzymes**	**Gene No**.
HBA	Phenylpropanoid biosynthesis	K10775	Phenylalanine ammonia-lyase (PAL)	1
		K01188	Beta-glucosidase (BGLU)	11
		K05349		
	Starch and sucrose metabolism	K01179	Endoglucanase (E3.2.1.4, EG)	1
		K01194	Alpha,alpha-trehalase (TREH)	1
		K13648	alpha-1,4-galacturonosyltransferase (GAUT)	1
		K16055	Trehalose 6-phosphate synthase/phosphatase (TPS)	1
	Circadian rhythm - plant	K16240	Protein suppressor of PHYA-105 1 (SPA1)	1
		K12120	Phytochrome A (PHYA)	1
Dactylorhin A	Starch and sucrose metabolism	K01193	Beta-fructofuranosidase (INV)	1
		K16055	Trehalose 6-phosphate synthase (TPS)	2
		K13648	Beta-amylase (E3.2.1.2, BAM)	2
		K00847	Fructokinase (E2.7.1.4, scrK)	2
		K01184	Polygalacturonase (E3.2.1.15, PG)	1
	Phenylpropanoid biosynthesis	K01904	4-coumarate-CoA ligase (4CL)	1
		K01188	Beta-glucosidase (BGLU)	6
		K00430	Peroxidase (E1.11.1.7, POD)	1
		K00083	Cinnamyl-alcohol dehydrogenase (CAD)	1
	Alanine, aspartate and glutamate metabolism	K01953	Asparagine synthase (AsnB)	5
		K00261	Glutamate dehydrogenase (GLUD1-2)	1
Coelonin	Phenylpropanoid biosynthesis	K10775	Phenylalanine ammonia-lyase (PAL)	3
		K01904	4-coumarate-CoA ligase (4CL)	1
		K00430	Peroxidase (E1.11.1.7, POD)	1
	Starch and sucrose metabolism	K00695	Sucrose synthase (SUS)	1
		K13648	Alpha-1,4-galacturonosyltransferase (GAUT)	1
	Glycolysis/ Gluconeogenesis	K13951	Alcohol dehydrogenase 1/7 (ADH1/7)	3
		K01568	Pyruvate decarboxylase (PDC)	2
		K00850	6-phosphofructokinase 1 (PFK)	1
		K01623	Fructose-bisphosphate aldolase (ALDO)	1
Militarine	Phenylpropanoid biosynthesis	K10775	Phenylalanine ammonia-lyase (PAL)	5
		K00487	Trans-cinnamate 4-monooxygenase (CYP73A)	1
		K01904	4-coumarate-CoA ligase (4CL)	2
		K05349	Beta-glucosidase (BGLU)	2
	Starch and sucrose metabolism	K01087	Trehalose 6-phosphate phosphatase (otsB)	3
		K16055	Trehalose 6-phosphate synthase (TPS)	2
		K01193	Beta-fructofuranosidase (INV)	1
		K00847	Fructokinase (E2.7.1.4, scrK)	1
	Glycolysis/ Gluconeogenesis	K00850	6-phosphofructokinase 1 (PFK)	1
		K00134	Glyceraldehyde-3-phosphate dehydrogenase (GAPDH)	2
		K08679	UDP-glucuronate 4-epimerase (GAE)	1
		K01689	Enolase (ENO)	1
		K01623	Fructose-bisphosphate aldolase (ALDO)	3
		K00873	Pyruvate kinase (PK)	2
		K00627	Dihydrolipoamide acetyltransferase (DLAT)	1
		K01568	Pyruvate decarboxylase (PDC)	6
		K18857	Alcohol dehydrogenase 1 (ADH1)	5
		K13951	Alcohol dehydrogenase 1/7 (ADH1/7)	2

##### DEGs Analysis of Dactylorhin A

Three time points−18 Dpi, 36 Dpi, and 42 Dpi—were detected to have significant differences with Dactylorhin A content, and these time points were selected for further differential expression analysis. Pairwise comparisons of 18 Dpi vs. 36 Dpi and 36 Dpi vs. 42 Dpi found 860 and 579 DEGs, respectively, with a total of 96 DEGs appearing to overlap. Among them, at the 18 Dpi vs. 36 Dpi time point, the number of upregulated genes was 554, and the number of downregulated genes was 306. At the 36 Dpi vs. 42 Dpi time point, the number of upregulated genes was 323, and the number of downregulated genes was 256.

The functional clustering arrays of GO and KEGG on the DEGs imply a mechanism for explaining how Dactylorhin A was biosynthesized. GO function analysis found that, at the 18 Dpi vs. 36 Dpi time point, 602 DEGs were annotated into the GO function database, with the majority of them enriched in hydrolase activity, O-acyltransferase activity, and carbohydrate metabolic process, and cysteine-type peptidase activity. In addition, at the 36 Dpi vs. 42 Dpi time point, 363 DEGs were mainly annotated in binding carboxylic acid metabolic process and oxidoreductase activity. While the KEGG pathway analysis found that, at the 18 Dpi vs. 36 Dpi time point, a total of 51 DEGs were significantly enriched in four pathways (*q-*value < 0.05), namely cyanoamino acid metabolism, starch and sucrose metabolism, phenylpropanoid biosynthesis, and alanine, aspartate, and glutamate metabolism pathways. In addition, the DEGs at the 36 Dpi vs. 42 Dpi were mainly annotated for the TCA cycle and starch and sucrose metabolism, among others (Supplementary Table S2). Selecting the metabolic pathways of starch and sucrose, which were closely related to the synthesis of Dactylorhin A, we found that there were 13 upregulated genes with significant differences, encoding UGP, BGLU, SPS, TPS, scrK, and 13 downregulated genes, encoding INV, SUS, TPS, BAM, and BGLU. Combined with the above analysis, 23 unigenes (related to 11 enzymes) were screened out as being involved in the synthesis of Dactylorhin A (Table 2).

##### DEGs Analysis of Coelonin

Based on the change in the content of Coelonin, we found that its content increased slowly from 3 Dpi to 30 Dpi and to 33 Dpi, remained relatively stable, and then increased exponentially before reaching its peak at 39 Dpi. Therefore, a pairwise comparison of the DEGs of 3 Dpi, 30 Dpi, 33 Dpi, and 39 Dpi was made (3 Dpi vs. 30 Dpi, 30 Dpi vs. 33 Dpi, 33 Dpi vs. 39 Dpi); there were 2,052, 1,461, and 849 DEGs, respectively. Among them, in the 3 Dpi vs. 30 Dpi time point, the number of upregulated genes was 1,162, and the number of downregulated genes was 890. At the 30 Dpi vs. 33 Dpi time point, the number of upregulated genes was 902, and the number of downregulated genes was 559. At the 33 Dpi vs. 39 Dpi time point, the number of upregulated genes was 316, and the number of downregulated genes was 533.

The functional clustering arrays of GO and KEGG on the DEGs imply a mechanism for explaining how Coelonin was biosynthesized. Through the comparative analysis of the GO database, the unigenes annotated by the GO can be divided into three categories: biological process, molecular function, and cellular component. At the 3 Dpi vs. 30 Dpi time point, DEGs were mainly involved in the metabolic process, cellular process, and organic substance metabolic process in biological processes; hydrolase activity, catalytic activity, and binding in molecular function. In the suspension cells of the 30 Dpi vs. 33 Dpi and 33 Dpi vs. 39 Dpi, DEGs were significantly enriched in the plant-type cell wall organization, plant-type cell wall organization, cell wall organization, and external encapsulating structure organization in biological processes; significantly enriched molecular functions were the structural constituents of the cell wall, such as structural molecule activity and others. The cell component that was significantly enriched was the histone deacetylase complex. Among them, the unigenes that are more abundant in biological processes may be related to the pathway of Coelonin synthesis concerned in this study.

KEGG enrichment results showed that, in comparison with 3 Dpi, 53 DEGs were involved in the metabolic pathways of starch and sucrose metabolism (ko00500) in 30 Dpi; 40 DEGs were enriched in the phenylpropanoid biosynthesis pathway (ko00940). At the 30 Dpi vs. 33 Dpi time point, there were 40 DEGs enriched in starch and sucrose metabolic pathways (ko00500). At the 33 Dpi vs. 39 Dpi time point, DEGs were enriched in the cyanoamino acid metabolism pathway (ko00460) (Supplementary Table S2).

We selected the phenylpropanoid biosynthesis, phenylalanine metabolism, and cyanoamino acid metabolism pathways, which were closely related to the synthesis of Coelonin. And according to the correlation analysis between gene expression and Coelonin content, the differential genes related to Coelonin metabolism were found. The results showed that the key regulatory genes mainly encoded PAL, 4CL, POD, SUS, GAUT, ADH, PDC, PFK, and ALDO. Finally, 14 unigenes (9 enzymes) were identified as potential Coelonin synthesis candidates (Table 2).

##### DEGs Analysis of Militarine

According to the change in the content of Militarine, it was found that from 3 Dpi to 30 Dpi, its content increased slowly and then increased exponentially before reaching its peak at 39 Dpi and then dropping sharply. Pairwise comparison of the DEGs of 3 Dpi, 30 Dpi, 39 Dpi, and 42 Dpi (3 Dpi vs. 30 Dpi, 30 Dpi vs. 39 Dpi, 39 Dpi vs. 42 Dpi) showed that there were 2052, 652, and 589 DEGs, respectively, and 1825, 496, and 410 DEGs appeared in only one DEG set, respectively; 33, 48, and 182 DEGs appeared in two DEG sets, respectively, and a total of 12 DEGs appeared in 3 DEG sets. Among them, at the 30 Dpi vs. 39 Dpi time point, the number of upregulated genes was 296, and the number of downregulated genes was 356; at the 39 Dpi vs. 42 Dpi time point, the number of upregulated genes was 387, and the number of downregulated genes was 202.

According to GO analysis, 1,344 DEGs were annotated into the GO database at 3 Dpi vs. 30 Dpi time point, with the majority of them enriched in hydrolase activity, asparagine synthase (glutamine-hydrolyzing) activity, catalytic activity, single-organism metabolic process, and carbohydrate metabolic process, among others. At the 30 Dpi vs. 39 Dpi time point, 476 DEGs were significantly enriched in cysteine-type peptidase activity, covalent chromatin modification, and histone modification. The KEGG pathway analysis of DEGs at three time points found that a total of 136 DEGs were significantly enriched in five pathways at 3 Dpi vs. 30 Dpi time point, namely starch and sucrose metabolism, phenylpropanoid biosynthesis, glycolysis/gluconeogenesis, photosynthesis, and phenylalanine metabolism pathways; at the 30 Dpi vs. 39 Dpi time point, a total of 30 DEGs were significantly enriched in Spliceosome; at the 39 Dpi vs. 42 Dpi time point, DEGs were mainly enriched in plant hormone signal transduction and phenylpropanoid biosynthesis (Supplementary Table S2). Based on the above analysis, 41 unigenes (18 key enzymes) of the main regulatory genes were screened out, which may be involved in the synthesis of Militarine, as shown in Table 2.

### Expression Analysis of Related Genes Regulating the Synthesis of Phenolic Acid Derivatives

**Figure 5:**
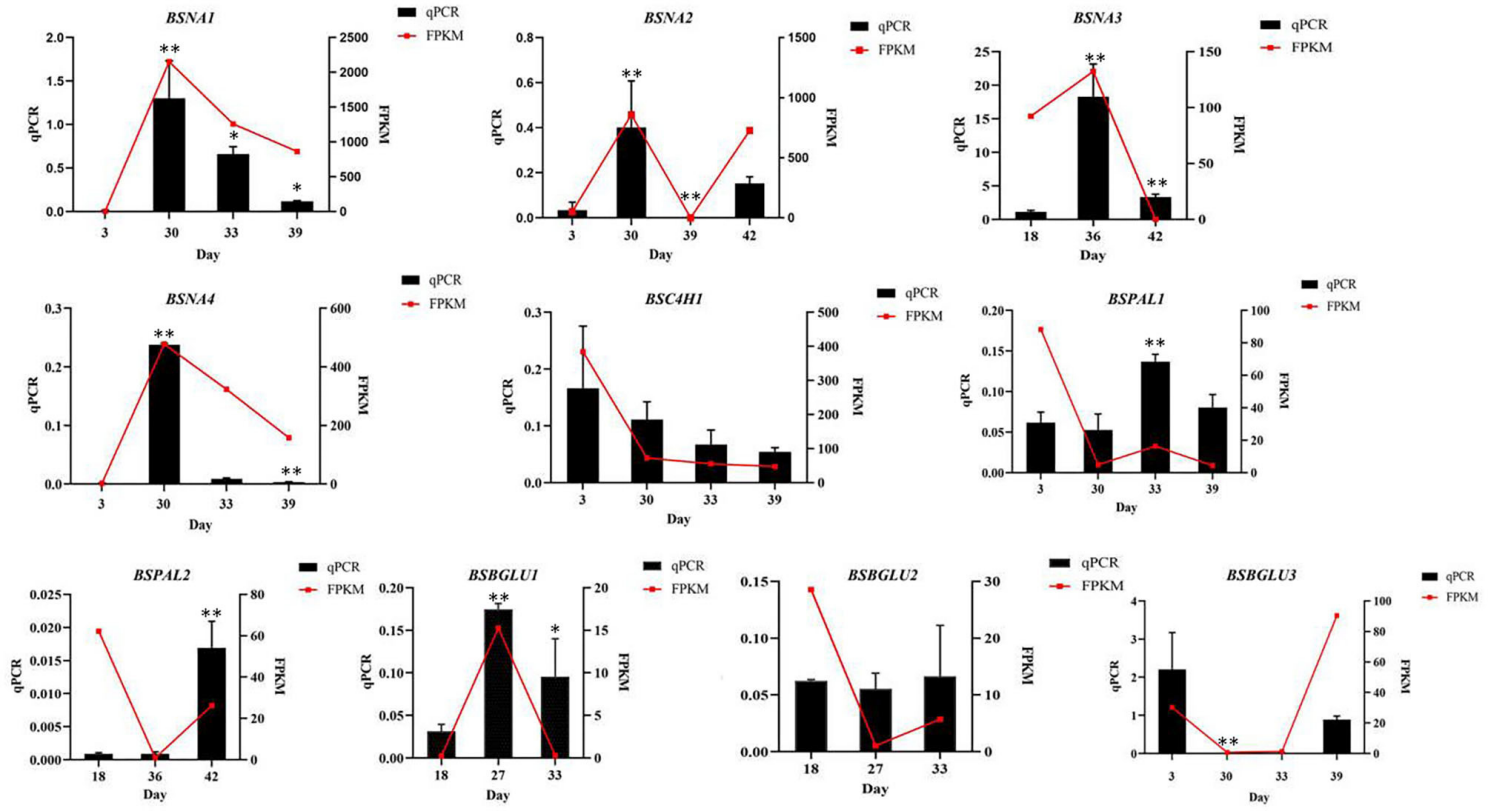
Comparison of expression of 10 genes obtained by qPCR analysis and by RNA-seq (FPKM values). * Represents the significant difference between the two groups compared with the previous group at a level of *P* < 0.05. ** represents the significant difference at a level of *P* < 0.01.

In order to verify the accuracy of gene expression in the transcriptome sequencing results, 10 DEGs were randomly selected from samples of four periods and verified by qPCR, in which the β*-actin* was applied as the internal reference gene. The results showed that the relative gene expression levels were consistent with the transcriptome sequencing results, indicating that the data obtained by transcriptome sequencing were accurate and reliable (Figure 5).

## Discussion

The secondary metabolites were produced by plants during their growth and development, as well as to defend against environmental stresses. In addition, the synthesis and accumulation of these metabolites can be significantly improved through suspension cell culture (Yang et al., 2018; Isah, 2019; Jiang et al., 2020). The secondary metabolites in suspension cells provide the material basis for their growth and reproduction. We analyzed the DEGs in different growth stages and found that many genes involved in the biosynthesis of secondary metabolites expressed significant differences in different growth stages. Moreover, the content of secondary metabolites also changed significantly, indicating that, with the change in suspension cell morphology and continuous growth and development, the synthesis of secondary metabolites of suspension cells is regulated by a variety of genes.

As time goes on, cultured suspension cells synthesize and accumulate secondary metabolites through different metabolic processes, thereby improving their ability to synthesize and accumulate HBA, Dactylorhin A, Militarine, and Coelonin in suspension cells of *B. striata* (Pan, 2019). This study found that the contents of HBA at 18 Dpi, 27 Dpi, and 33 Dpi, Dactylorhin A at 18 Dpi, 36 Dpi, and 42 Dpi, Coelonin at 3 Dpi, 30 Dpi, 33 Dpi, and 39 Dpi, and Militarine at 3 Dpi, 30 Dpi 39 Dpi, and 42 Dpi were significantly different by HPLC, respectively. It was found that, compared with the high-efficiency culture lag zone, the content of the four metabolites in the exponential phase increased significantly; the content of Dactylorhin A, Militarine, and Coelonin decreased significantly in the declining phase, while HBA slowly increased. According to the chemical structures of these four compounds (Supplementary Figure 1), the main structures of Dactylorhin A and Militarine were the same, which implies that they may have connections in the biosynthesis process. The increase in Militarine content resulted in a reduction of Dactylorhin A synthesis. HBA was simple in structure, which could be an integral part of forming Militarine and Coelonin. Therefore, HBA contributes to the increase of Militarine and Coelonin.

Genes involved in various metabolic processes of cultured suspension cells are critical in the process of the synthesis of secondary metabolites. The metabolism of phenolic acid compounds was a relatively complicated process. The shikimic acid pathway, the phenylpropanoid metabolic pathway, and the flavonoid metabolic pathway were the main pathways for the synthesis of phenolic acid compounds (Saxe et al., 2021). Among them, phenylalanine and tyrosine are direct precursors of phenolic acid biosynthesis, which are converted from glucose into a multistep metabolism via the shikimic acid pathway and then into the phenylpropanoid metabolic pathway (Feduraev et al., 2020; El-Shora et al., 2021). Additionally, phosphoenolpyruvate and erythrose-4-phosphoric acid were the main precursor substance for the synthesis of phenolic acid compounds (Deng et al., 2020). Phenylalanine was deaminated and transformed into trans-cinnamic acid by phenylalanine ammonia-lyase (PAL) (Trivedi et al., 2022). Subsequently, phenolic acids such as trans-cinnamic acid, p-coumarate, and ferulic acid are formed under the action of cinnamate 4-hydroxylase (C4H), 4-coumaratecoa-ligase (4CL), O-methyltransferase (OMT), and other enzymes catalyzed further into caffeic acid and chlorogenic acid (Wohl and Petersen, 2020). HBA was one of the main components of phenolic compounds and the main precursor of aromatic compounds (Zhang Z. L. et al., 2020). Former studies have confirmed that Dactylorhin A and Militarine were glycoside compounds isolated from plants, which were produced by the loss of water from the hydroxyl amino thiol group of monosaccharides or oligosaccharides and the hemiacetal hydroxyl group of another molecule. With the growth and development of suspension-cultured cells, the expression levels of genes involved in the metabolism of starch and sucrose and the biosynthetic pathway of phenylpropanoid differed significantly. The differences in the expression of these genes may be the main reason for the significant differences in the content of glycosides in different periods. BGLU is a group of heterogeneous hydrolases that have the ability to hydrolyze and synthesize glycosidic bonds and have great application value in various fields (Santos et al., 2018; Sun et al., 2020). The enhanced expression of genes involved in BGLU may promote the synthesis of carbohydrates and provide energy for the growth and development of suspension-cultured cells. At the same time, BGLU was regulated by a large number of genes at different time points and was involved in the synthesis of phenolic acids, which indicated that BGLU may be involved in the synthesis of phenolic acids and other substances and maintaining cell morphology during the development of *B. striata*. Coelonin was a dihydrophenanthrene compound. The methoxy and hydroxyl groups were the main substituents on the benzene ring of the dihydrophenanthrene compound. From the point of view of molecular structure, phenolic acid and its derivatives can be obtained after the oxidation of dihydrophenanthrene compounds.

In this study, we detected several interesting co-changes between gene expression and chemical content, which may infer the genes' function. The expression of i1_LQ_BSbp1_C76772/f1p0 increased steadily from 0 to 8 Dpi but decreased dramatically later, which was positively correlated with the content changes of Dactylorhin A. The expression of i3_LQ_BSbp1_C942 /f1p19 was continuously downregulated from 0 to 36 Dpi but upregulated from 36 Dpi to 39 Dpi and then downregulated again, which was positively correlated with the content change of Dactylorhin A. The expression of i3_LQ_BSbp1_C5868/f1p2 was continuously downregulated at the stage of 0–18 Dpi but then upregulated, which showed a positively significant correlation with the content of Militarine. Based on these results, we predict that the TPS plays a very important role in plant metabolic regulation. In addition, we also speculated that these genes are closely related to the biosynthesis of Dactylorhin A and Militarine. Similar to this analysis and speculation, we built the relationship between genes i2_HQ_BSbp1_C5228/f3p1 and Dactylorhin A and gene i2_LQ_BSbp1_C117598/f1p12 and Militarine. In addition, the INV may be key to the synthesis of Dactylorhin A and Militarine. Therefore, during the growth of *B. striata* suspension culture cells, with the passage of time, the expression of key enzyme genes regulating the synthetic biological pathway is different in different periods, and through interaction, the content of phenolic acid derivatives is different in different periods.

In different growth periods, the metabolic pathways involved in the synthesis of these four secondary metabolites of DEGs mainly include phenylpropanoid biosynthesis, phenylalanine metabolism, cyanoamino acid metabolism, and starch and sucrose metabolism. The results of the enrichment of metabolic pathways indicated that carbohydrate metabolism was needed as a basis during development, and hormones and some secondary metabolites were required to co-regulate. According to the KEGG pathway, GO enrichment analysis, and correlation analysis of DEGs, the final screening of enzymes involved in the synthesis of HBA, Dactylorhin A, Militarine, and Coelonin were 8, 11, 18, and 9, respectively, and the related unigenes were 18, 23, 41, and 14, which led to the speculation that these genes which were screened out may be the key regulators of phenolic acid derivatives. Therefore, the differential expression of key enzyme genes in the biosynthetic pathway at different periods may ultimately lead to differences in the content of phenolic acid derivatives at different periods.

At present, four phenolic acid derivatives of secondary metabolites have been isolated and identified, but the metabolic mechanism of their synthesis and accumulation has not been studied. It was found that the content of Dactylorhin A, Millitarine, and other compounds in *B. striata* was higher than the cultivated ones (Wang et al., 2014). In this study, gene expression profiles of suspension cells at different growth stages were obtained through the transcriptome method, and a total of 17,500 DEGs were screened. The preliminary screening showed that the genes of PAL, 4CL, BGLU, INV, TPS, BAM, and PDC may regulate the key enzyme of phenolic acid derivatives. Through the gene identification and regulatory mechanism exploring of four main secondary metabolites, the key regulated genes in this study could be overexpressed or silenced in the future to further verify their functions in the biosynthesis of four phenolic acid derivatives in *B. striata*. Thus, the metabolic mechanism of synthesis and accumulation of the four secondary metabolites will be thoroughly explained. This provided data for an in-depth study of the synthesis and regulation mechanisms of phenolic acid derivatives in suspension cells of *B. striata*.

## Data Availability Statement

The datasets presented in this study can be found in online repositories. The names of the repository/repositories and accession number(s) can be found below: National Center for Biotechnology Information (NCBI) BioProject database under accession numbers PRJNA807395 and PRJNA807396.

## Author Contributions

DX conceived, supervised, wrote, and reviewed the manuscript. HL, CH, WW, and LL originally wrote and reviewed the draft. HL, CH, QL, MW, SX, JS, and YH conducted the experiments and carried out the analysis. DX founded and administrated the project. All authors read and approved the final version.

## Funding

This research was financially supported by the National Natural Science Foundation of China (31560079, 31960074), the Science and Technology Department Foundation of Guizhou Province (No. [2017]5733-050, [2019]-027, [2019]5657), the Special Joint Bidding Project of Zunyi Sci & Tech Bureau and Zunyi Medical University (ZSKHHZ-2020-91), the Honghuagang Sci & Tech Project (ZHKHNZT [2020]04), and the Program for Excellent Young Talents of Zunyi Medical University (15zy-004).

## Conflict of Interest

The authors declare that the research was conducted in the absence of any commercial or financial relationships that could be construed as a potential conflict of interest.

## Publisher's Note

All claims expressed in this article are solely those of the authors and do not necessarily represent those of their affiliated organizations, or those of the publisher, the editors and the reviewers. Any product that may be evaluated in this article, or claim that may be made by its manufacturer, is not guaranteed or endorsed by the publisher.

## Supplementary Material

Supplementary Figure 1 Structure of four phenolic acid derivatives.

Supplementary Table S1 Correlation between different phenolic acid derivatives.

Supplementary Table S2 Annotation of DEGs.

Supplementary Table S3 Main experimental reagent.

Supplementary Table S4 Parameter values for HPLC assay.

Supplementary Table S5 cDNA synthesis.

Supplementary Table S6 qPCR reaction system.
