# Supplementary figures and images for: Genome-Wide Identification of Genes Related to Biosynthesis of Phenolic Acid Derivatives in Bletilla striata at Different Suspension Culture Stages
Source: Front Plant Sci. 2022 Jun 17;13:875404. doi: 10.3389/fpls.2022.875404 (PMC9247868; doi:10.3389/fpls.2022.875404)

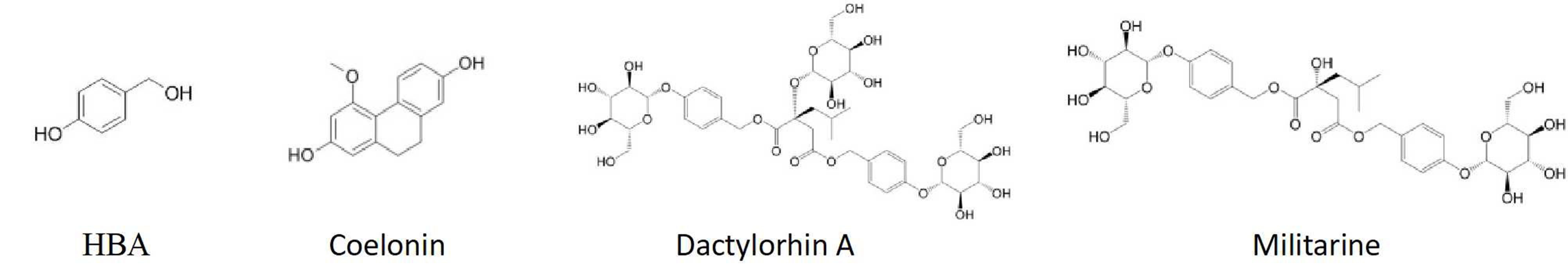

Supplement: Supplementary Figure 1 — Structure of four phenolic acid derivatives. [file Image_1.JPEG]
